# Detection, Isolation and Characterization of Principal Synthetic Route Indicative Impurities in Verapamil Hydrochloride

**DOI:** 10.3797/scipharm.1101-19

**Published:** 2011-05-08

**Authors:** Viswanathan Srinivasan, Hariharan Sivaramakrishnan, Balakrishnan Karthikeyan

**Affiliations:** 1 Analytical Development Laboratory, Piramal Healthcare Ltd, Ennore, Chennai 600 057, India; 2 Department of Chemistry, Annamalai University, Annamalainagar 608 002, India; 3 Research and Development Laboratory, Piramal Healthcare Ltd, Ennore, Chennai 600 057, India

**Keywords:** Verapamil hydrochloride, NMR, HPLC, MS, Impurity Analysis

## Abstract

Two unknown impurities were detected in verapamil hydrochloride bulk drug using isocratic reversed-phase high performance liquid chromatography (HPLC). These impurities were isolated by preparative HPLC. Spectral data for the isolated impurities were collected. Based on the spectral data derived from two-dimensional nuclear magnetic resonance (2D-NMR) spectroscopy and mass spectrometry (MS), impurity-1 and impurity-2 were characterized as 2-(3,4-dimethoxyphenyl)-3-methylbut-2-enenitrile and 2-(3,4-dimethoxyphenyl)-2-isopropyl-3-methylbutanenitrile, respectively.

## Introduction

Verapamil hydrochloride, chemically known as 2-(3,4-dimethoxyphenyl)-5-{[2-(3,4-di-methoxyphenyl)ethyl](methyl)amino}-2-(1-methylethyl)pentanenitrile [1], is an oral and intravenous calcium-channel blocking (CCB) agent. It is administered in the treatment of angina, hypertension, and supraventricular tachyarrhythmia. Verapamil hydrochloride is considered as a class IV antiarrhythmic agent and it is more effective than digoxin for controlling ventricular rate in patients with atrial fibrillation.

Its principal physiological action is to inhibit the transmembrane influx of calcium ions into the heart and vascular smooth muscle cells. It improves the relationship between oxygen supply and consumption in the myocardium because oxygen demand is lowered directly as a result of the effect on the energy consuming metabolic process of the myocardial cells and indirectly due to a reduction of the afterload. Verapamil hydrochloride inhibits the influx of extracellular calcium across the myocardial and vascular smooth muscle cell membranes. It exerts its activity at the membrane surface of arterial smooth muscle cells and within conductile and contractile tissue in the myocardium. Calcium channels in myocardial and vascular smooth muscle cell membranes are selective and allow a slow inward flow of calcium, which contributes to excitation–contraction coupling and electrical discharge of conduction cells (plateau phase of the action potential) in the heart and vasculature. Verapamil hydrochloride inhibits this influx, possibly by deforming the channel, or by inhibiting ion-control gating mechanisms. Verapamil hydrochloride may also interfere with the release of calcium from the sarcoplasmic reticulum inside the cell. The decrease in intracellular calcium inhibits the contractile processes of the myocardial smooth muscle cells, resulting in dilation of the coronary and systemic arteries. These actions increase oxygen delivery to myocardial tissue and decrease total peripheral resistance, systemic blood pressure and afterload. This reduction in myocardial oxygen demand, cardiac workload and vascular tone is believed to be responsible for the drug’s beneficial effects in angina and its antihypertensive activities. Inhibition of calcium-mediated smooth muscle contraction is also thought to be the explanation for verapamil hydrochloride’s action in the prevention and treatment of migraine [2–4].

Verapamil hydrochloride has also been described as an inhibitor of P-glycoprotein and is able to improve the response to chemotherapy by reducing the resistance of cancer cells against antineoplastic agents [5].

Recent studies on verapamil hydrochloride have revealed that a high dose of the drug is positive in the treatment of cluster headache. The dose of verapamil hydrochloride used for cluster headache is approximately double the dose used in cardiovascular disease, most likely because verapamil hydrochloride is a substrate for the efflux transporter P-glycoprotein in the blood–brain barrier. Access of verapamil hydrochloride to the central nervous system is therefore limited. The effect of verapamil hydrochloride in cluster headache most likely takes place in the hypothalamus [6].

Verapamil hydrochloride was synthesized in our laboratory, and when a sample was subjected to high performance liquid chromatography (HPLC) analysis, we noted the presence of two unknown impurities which were isolated by preparative HPLC [7, 8]. The structural elucidation of these impurities is the objective of this work.

The different analytical techniques reported so far for the determination of this drug along with associated impurities are capillary electrophoresis [9, 10], spectrophotometry [11, 12], HPLC [13, 14], atomic emission spectrometry [15], and the Rayleigh scattering method [16].

During quality control (QC) release analysis of the verapamil hydrochloride sample, two unknown impurities were observed at 0.68 RRT (relative retention time) and 1.07 RRT at the 0.10% level when the sample was analyzed using chromatographic conditions published in the British Pharmacopoeia (BP) monograph for verapamil hydrochloride [17]. As per the regulatory guidelines, pharmaceutical studies using a sample of isolated impurities can be considered for safety assessment. Also, knowledge of the structure and source of the novel impurities is necessary to develop a more robust synthetic process. It is therefore essential to isolate and characterize unidentified impurities present in the drug sample. A sample was taken for the isolation of unknown impurities using preparative HPLC and the isolated impurities were characterized using mass spectrometry (MS), nuclear magnetic resonance (NMR) spectroscopy and infrared (IR) spectroscopy. To the best of our knowledge, this is the first report of these impurities detected at 0.68 RRT and 1.07 RRT.

## Experimental

### Chemicals

Acetonitrile, dipotassium hydrogen phosphate, formic acid and orthophosphoric acid, all HPLC grade, were purchased from Merck (Germany).

### Preparative high performance liquid chromatography (Prep HPLC)

Shimadzu preparative HPLC equipment was used with an LC-8A pump, SCL-10A VP system controller, SPD-10AVP UV-VIS detector, FRC-10A fraction collector and Rheodyne injector with 5.0 mL loop (Shimadzu, Japan). The chromatographic separation was achieved on a Gemini C18 (Phenomenex, USA), 150 mm × 30.0 mm, 5 μm semi-preparative column using a mobile phase containing a mixture of water and acetonitrile (65:35, *v/v*). The flow rate of the mobile phase was 20.0 mL/min and the wavelength was monitored at 278 nm. The injection volume was 1000 μL. The test concentration for the analysis was 50 mg/mL. The mobile phase was used as diluent for the preparation of test solutions. The data were recorded using Shimadzu’s LCsolution software. The eluent was monitored at 278 nm and the fractions collected were injected into the analytical HPLC apparatus to confirm the retention times. The fractions collected from preparative HPLC were evaporated using a Rotavapor.

### High performance liquid chromatography (analytical)

The HPLC system was equipped with Quaternary gradient pumps, autosampler and autoinjector (Model LC2010CHT, Shimadzu, Japan) connected to a photodiode array detector controlled by LCsolution software (Shimadzu, Japan). A C18 column, ABZ+ 250 × 4.6 mm, 5 μm (Sigma–Aldrich) was used with a mobile phase consisting of a mixture of 6.97 g of dipotassium hydrogen phosphate in 1000 mL of water, pH adjusted to 7.2 with phosphoric acid. The eluent was monitored at 278 nm at a flow rate of 1.5 mL/min. The verapamil hydrochloride drug sample was dissolved in mobile phase as diluent after sonicating the sample for about 5 min; the sample was further filtered through a 0.2 μm syringe filter and then injected into the HPLC apparatus.

### Mass spectrometry

Direct ionization mass spectrometry was performed on a GCMS-2010 (Shimadzu, Japan) equipped with a DI source. The sample was introduced into the ion source with detector voltage 1.25 kV, detector temperature 300°C, ion source temperature 225°C and ionization energy 70 eV. The data were recorded using GC Solution software.

### High resolution mass spectrometry (HR-MS)

The high resolution mass spectrometer consisted of an Agilent 1200 series high performance liquid chromatography system and a micrOTOFQ mass spectrometer (high sensitivity orthogonal time-of-flight instrument, Bruker Daltonics, Germany). All samples were analyzed on the micrOTOFQ mass spectrometer equipped with an electrospray ionization (ESI) source for accurate mass values. An in-house compound was used as an internal reference, and was introduced via a six-port divert valve using the Hystar and micrOTOF control software. The mass range was calibrated with ESI tuning mix solution (Part No. G2421A) from Agilent Technologies using quadratic fit. The sample was dissolved in methanol, sonicated for 10 min and diluted further to obtain a concentration of 10 μg/mL. Then, using an autosampler, 5 μL of sample was introduced into the flow injection of 0.1% formic acid solution in water and acetonitrile (1:1) at a flow rate of 200 μL/min. The ESI tuning mix solution (1:10) in acetonitrile containing 10 μg/mL of in-house reference compound was infused via an infusion syringe to fill the 20 μL injection loop on a six-port divert valve. Using the micrOTOF control software, elution of the sample peak followed by that of the ESI tuning mix was programmed.

### NMR

The ^1^H, ^13^C, and DEPT (Distortionless Enhancement by Polarization Transfer) experi-ments were performed with an JEOL AL 300 MHz FT-NMR spectrometer. All 2D-NMR experiments, Correlation Spectroscopy (COSY), Heteronuclear Single Quantum Correlation (HSQC) and Heteronuclear Multiple-Bond Coherence (HMBC) were performed using a gradient probe on a Bruker Avance 500 MHz FT-NMR spectrophotometer equipped with a 5 mm ^1^H/^13^C/X (^15^N) three-channel triple resonance (TXI) probe; the pulse program used was employed from the pulse program library of Bruker. The data obtained were processed and analyzed using Topspin version 1.3 software (Bruker). CDCl_3_ was used as solvent.

The ^1^H and ^13^C chemical shift values were reported on the δ scale in ppm, relative to tetramethylsilane (TMS) (δ 0.00 ppm) as internal standard. The coupling constants were expressed in Hertz. The IR spectra were recorded in the solid state as KBr dispersion using a Perkin-Elmer Spectrum One FT-IR spectrophotometer.

## Results and Discussion

**Fig. 1.**
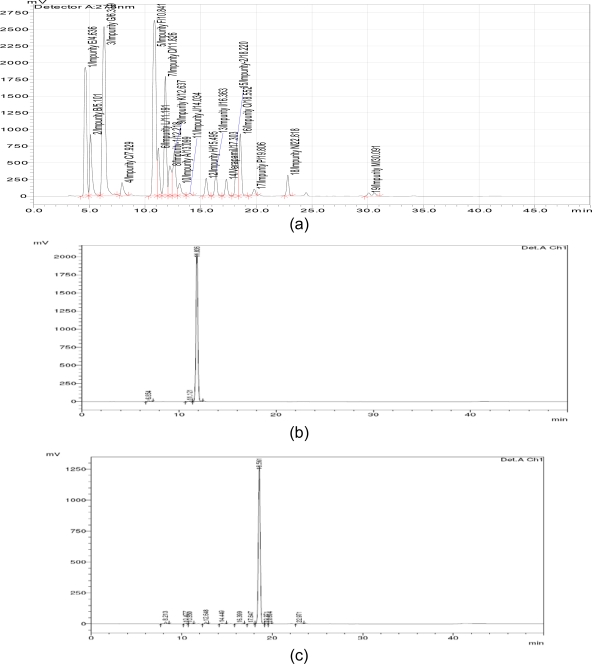
Typical chromatograms of (a) verapamil hydrochloride with impurities, (b) isolated impurity-1 and (c) isolated impurity-2.

**Fig. 2.**
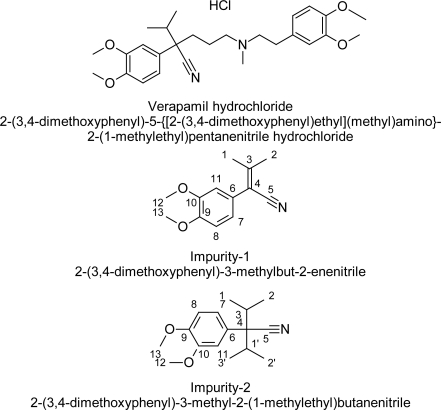
Chemical structures of verapamil hydrochloride and its impurities.

The isolated impurities were analyzed by HPLC and the HPLC purity was 99.2% for impurity-1 and 97.8% for impurity-2. The analytical HPLC chromatograms of verapamil hydrochloride along with impurities (including 16 impurities specified in BP) are shown in Fig. 1. Verapamil hydrochloride eluted at a retention time of 17.3 min, impurity-1 at 11.8 min and impurity-2 at 18.5 min. The structures of verapamil hydrochloride and the isolated impurities are shown in Fig. 2.

To obtain a preliminary structural insight, mass analysis was carried out on the isolated sample. The mass spectra of impurity-1 showed the molecular ions at *m*/*z* 217 and impurity-2 at *m*/*z* 261, respectively, whereas verapamil hydrochloride displayed molecular ions at *m*/*z* 454. The impurities were separated from verapamil hydrochloride by preparative HPLC so as to isolate larger amounts of impurity-1 and impurity-2 suitable for spectroscopic investigation. After isolation of impurity-1 and impurity-2, analytical HPLC chromatograms are shown in Fig. 1. The NMR data of the isolated impurities were collected and the structures of the impurities have been assigned with the help of COSY, HSQC and HMBC data.

### Structural elucidation of impurity-1

HR-MS data showed the mass of the molecular ion as a sodium adduct at *m*/*z* 240.0995 [(corrected *m*/*z* 217.1097) (calculated 217.1103 for C_13_H_15_NO_2_)], which corresponds to the molecular formula C_13_H_15_NO_2_.

**Fig. 3.**
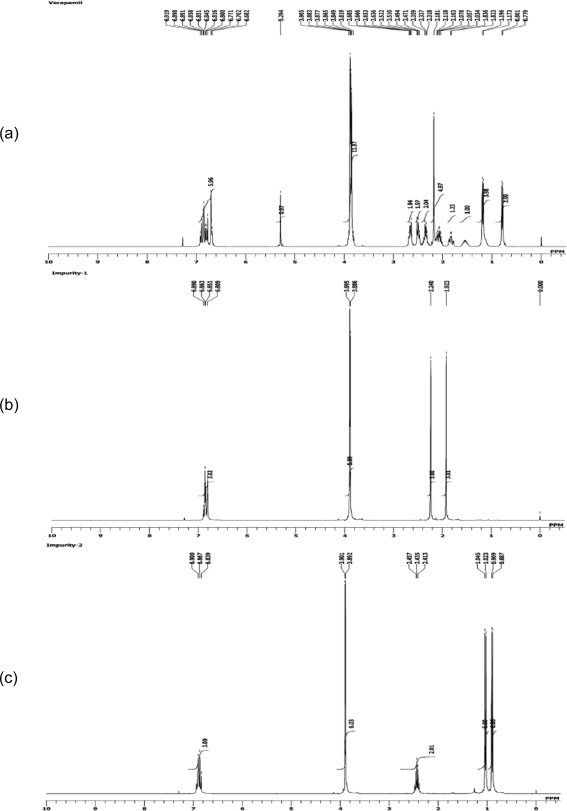
^1^H NMR Spectra of a) verapamil b) impurity-1 and c) impurity-2

The ^1^H NMR data for impurity-1 (Fig. 3b) were compared with those of verapamil hydrochloride (Fig. 3a). The ^1^H NMR spectrum shows that there are two methoxy functional groups, three aromatic protons and two methyl groups. The methyl signals are singlets which means that there is no proton on the neighboring carbon atom.

The ^13^C NMR data (Table 1) shows that there are three tertiary aromatic carbons and six quaternary carbons in the aromatic region which means that the phenyl ring is tri-substituted which accounts for three quaternary carbons and the presence of –CN accounts for another quaternary carbon. The peak at 118.98 ppm is due to the presence of –CN. The peak at 112.03 ppm could be due to the presence of a quaternary carbon attached to the –CN group.

From the DEPT 135 spectrum, it is clear that there are no –CH_2_ functional groups present in this moiety. From DEPT 135 and DEPT 90, it is also clear that there are three –CH groups in the aromatic region and two –CH_3_ groups present. No –CH group is present in the aliphatic region.

The HMBC spectrum shows connectivity between –CH_3_ and two quaternary carbons at 119 ppm and 110.5 ppm.

**Fig. 4.**
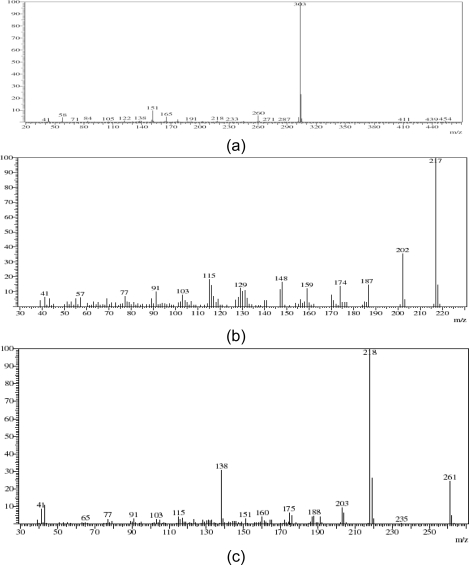
Mass spectra of a) verapamil hydrochloride b) impurity-1 and c) impurity-2

The mass data for verapamil hydrochloride (Fig. 4a) and impurity-1 (Fig. 4b) were compared. The data indicate that the molecular ion of verapamil hydrochloride is at *m*/*z* 454[(M)^+^] and the mass data for impurity-1 displayed a molecular ion at *m*/*z* 217[(M)^+^]. Thus impurity-1 has 237 a.m.u. less than that of the molecular ion of verapamil hydrochloride and the mass of the impurity is nearer to the mass of one of the intermediates, (2*RS*)-2-(3,4-dimethoxyphenyl)-3-methylbutanenitrile.

Further, the fragmentation pattern of impurity-1 at *m*/*z* 202 indicates the cleavage of one –CH_3_ group, *m*/*z* 187 indicates the cleavage of a further –CH_3_ group, *m*/*z* 174 indicates the cleavage of an isopropyl group and *m*/*z* 148 indicates the cleavage of a –CN group.

From the ^13^C NMR data, it was inferred that there are two additional quaternary carbons, which may be due to an isopropene group. Based on the 2D-NMR and MS data, the structure of impurity-1 is characterized as 2-(3,4-dimethoxyphenyl)-3-methylbut-2-enenitrile.

### Structural elucidation of impurity-2

HR-MS data showed the exact mass of the molecular ion as a sodium adduct at *m*/*z* 284.1611 [(corrected *m*/*z* 261.1713) (calculated 261.1729 for C_16_H_23_NO_2_)], which corresponds to the molecular formula C_16_H_23_NO_2_.

The ^1^H NMR data of impurity-2 (Fig. 3c) were compared with those of verapamil hydrochloride (Fig. 3a). This impurity has two methoxy (–OCH_3_) functional groups which are present in a similar region to verapamil hydrochloride. In the aromatic region, there are three protons and two sets of doublets around 1.0 ppm which could indicate a methyl group. There is another septet at 2.43 ppm which could be due to a –CH group. The presence of these two sets of peaks in the aliphatic region lead us to believe that there could be two isopropyl groups present in this impurity.

The ^13^C NMR data (Table 1) show that there are five quaternary carbons including a –CN group; the peak at 121.97 ppm indicates the presence of a –CN functional group and there is one quaternary carbon present in the aliphatic unit. Considering the mass data, there are two –OCH_3_ functional groups, two sets of isopropyl groups, and –CN with a quaternary carbon. From the ^1^H and ^13^C NMR data, there are three –CH groups and three quaternary carbons present in the moiety.

From the DEPT 135 spectrum, it is clear that there is no –CH_2_ group present in this molecule.

From the COSY NMR data, both –CH groups are present in the same position which indicates that they are in the same chemical and magnetic environment.

The HMBC spectrum shows the connectivity of –CH (isopropyl) with the quaternary aromatic and aliphatic carbon and also –CN. Both sets of –CH_3_ (isopropyl) show connectivity to the aliphatic quaternary carbon and –CN. Both the methoxy functional groups show direct connectivity to the phenyl ring.

The mass data for verapamil hydrochloride (Fig. 4a) and impurity-2 (Fig. 4c) were compared. The data indicate that the molecular ion peak of verapamil hydrochloride is at *m*/*z* 454[(M)^+^] and the molecular ion peak of impurity-2 at *m*/*z* 261[(M)^+^]. Thus, impurity-2 has 193 a.m.u. less than that of the molecular ion of verapamil hydrochloride. Further fragmentation patterns of impurity-2 at *m*/*z* 235, 218 and 175 indicate the cleavage of a –CN group, one isopropyl group followed by one more isopropyl group. The fragmentation due to *m*/*z* 138 indicates the presence of a dimethoxybenzene analog. This observation is also supported by proton NMR, where two sets of peaks were observed in the aliphatic region, and which indicates the presence of two isopropyl groups in this impurity.

Based on the 2D-NMR and MS data, the structure of impurity-2 is characterized as 2-(3,4-dimethoxyphenyl)-2-isopropyl-3-methylbutanenitrile.

## Conclusion

In the present study, two unknown impurities, impurity-1 and impurity-2, were detected by HPLC of verapamil hydrochloride bulk drug. The source for the formation of impurity-1 was due to contamination by acetone which was used as the cleaning solvent. The source for the formation of impurity-2 could be attributed to the addition of excess base (NaNH_2_) to the reaction mass. The attempt to isolate these impurities has been successful. They were isolated by preparative HPLC and characterized by spectroscopic studies. The structures of these impurities have not been reported previously in the literature. This study highlights the importance of impurity profiling of a drug substance.

Based on the above spectral data, the molecular formula for impurity-1 was confirmed as C_13_H_15_NO_2_ and the corresponding structure was characterized as 2-(3,4-dimethoxy-phenyl)-3-methylbut-2-enenitrile. Similarly, for impurity-2, the molecular formula was confirmed as C_16_H_23_NO_2_ and the corresponding structure was characterized as 2-(3,4-di-methoxyphenyl)-2-isopropyl-3-methylbutanenitrile.

## Figures and Tables

**Fig. 5.**
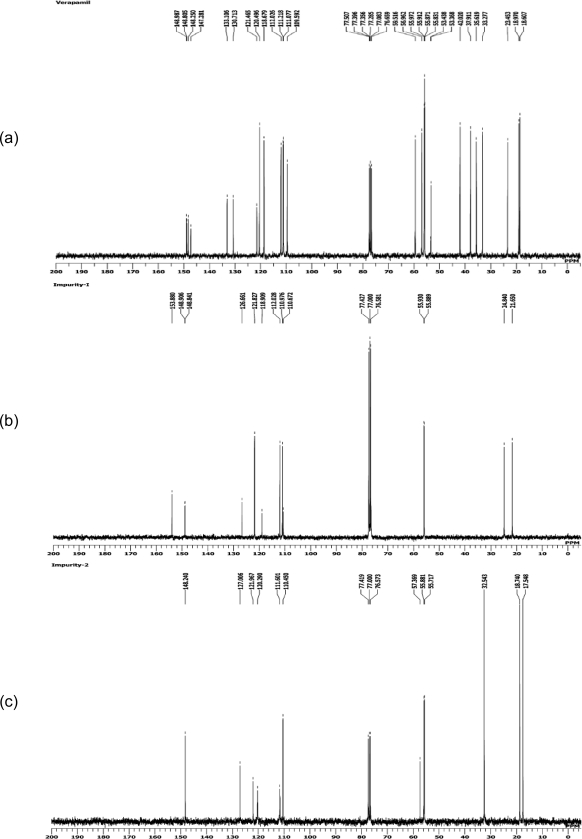
^13^C NMR Spectra of a) verapamil b) impurity-1 and c) impurity-2

**Tab. 1.** ^1^H and ^13^C NMR assignments for impurity-1

**S.No**	**Shift (ppm)**	**Multiplicity**	**No.of H**	**Assignment**
1	0.89–0.91	Singlet	3	1 (2)
2	1.03–1.05	Singlet	3	2 (1)
3	3.90–3.91	Singlet	6	12,13
4	6.84–6.91	Multiplet	3	7,8,11

**S.No**	**Shift (ppm)**	**Carbon**	**Assignment**
1	21.65	–CH_3_	C-1
2	24.84	–CH_3_	C-2
3	55.89	–CH_3_	C-12*
4	55.93	–CH_3_	C-13*
5	110.67	–CH	C-11
6	110.97	–CH	C-8
7	112.03	-C	C-4
8	118.98	-C	C-5
9	121.83	–CH	C-7
10	126.66	-C	C-6
11	148.84	-C	C-10*
12	148.90	-C	C-9*
13	153.88	-C	C-3

* Tentative assignment

**Tab. 2.** ^1^H and ^13^C NMR assignments for impurity-2

**S.No**	**Shift (ppm)**	**Multiplicity**	**No.of H**	**Assignment**
1	0.89–0.91	Doublet	6	1,1’ (2,2’)
2	1.02–1.05	Doublet	6	2,2’(1,1’)
3	2.41–2.46	Septet	2	3,3’
4	3.89 & 3.90	Singlets	6	12,13
5	6.81–6.89	Multiplet	3	7,8,11

**S.No**	**Shift (ppm)**	**Carbon**	**Assignment**
1	17.55	–CH_3_	C-1,1’
2	18.74	–CH_3_	C-2,2’
3	32.54	–CH	C-3,3’
4	55.72	–CH_3_	C-12*
5	55.88	–CH_3_	C-13*
6	57.37	-C-	C-4
7	110.45	–CH	C-11
8	111.60	–CH	C-8
9	120.29	–CH	C-7
10	121.97	–CN	C-5
11	127.01	-C-	C-6
12	148.24	-C-	C-9,10

* Tentative assignment
